# Response Surface Optimized Infrared-Assisted Extraction and UHPLC Determination of Flavonoid Types from Flos Sophorae

**DOI:** 10.3390/molecules22061000

**Published:** 2017-06-15

**Authors:** Qianqian Mou, Jingxia He, Rongli Yin, Bin Yang, Meihong Fu, Jing Fang, Hua Li

**Affiliations:** 1 Institute of Chinese Materia Medica, China Academy of Chinese Medical Sciences, Beijing 100700, China; qqmou22@163.com (Q.M.); hejingxia@163.com (J.H.); ybinmm@126.com (B.Y.); fu00126@sina.com (M.F.); j_fang0817@sina.com (J.F.); 2 College of Pharmacy, Chengdu University of Traditional Chinese Medicine, Chengdu 611137, China; yinronglili@163.com; 3 State Key Laboratory of Dao-di Herbs, China Academy of Chinese Medical Sciences, Beijing 100700, China

**Keywords:** antioxidant activity, flavonoid, infrared-assisted extraction, Flos sophorae, ultra-high performance liquid chromatography

## Abstract

Single-factor experiment and Box-Behnken design were applied to optimize the infrared-assisted extraction (IRAE) of rutin, quercetin, kaempferol, and isorhamnetin from Flos sophorae. Four factors (extract solvent, solid-liquid ration, extraction time, infrared power) affecting the extraction yield of flavonoids were studied. Under optimized conditions the extraction yield was 33.199 ± 0.24 mg/g, which substantially improved, compared with heating reflux extraction (HRE) and ultrasonic-assisted extraction (UAE), while extraction time was only 9 min. The eluents were rich in 2,2-diphenyl-1-picrylhydrazyl (DPPH) and 2,2′-azobis (2-methyl-propionamidine) dihydrochloride radical scavenging potential (IC_50_ of DPPH: 53.44 ± 0.01 μg/mL, oxygen radical absorbance capacity (ORAC): 3785.83 ± 52 μmol/g) than the extracts obtained by HRE and UAE. In addition, an ultra-high performance liquid chromatography method was optimized for the identification and quantification of the tested flavonoids, and the method was validated based on its correlation coefficient (*r*), reproducibility (RSD, *n* = 5), and recovery values, which were 0.9994–0.9998, 0.74–1.83%, and 97.78–102.94%, respectively. These results confirmed that high extraction yield of flavonoids results in stronger antioxidant values and response surface methodology optimization of IRAE is a promising alternative to traditional extraction techniques for flavonoids from medicinal plants.

## 1. Introduction

Natural antioxidants derived from medicinal plants have attracted considerable interest during the last decade from researchers working in a variety of different fields because of their beneficial effects for human health. For example, natural antioxidants have been reported to exhibit protective effects against chronic degenerative diseases, such as cancer and cardiovascular diseases, because of their antioxidant activity [1]. Moreover, natural antioxidants rarely pose mutagenic/genotoxic risks to human health [2], whereas synthetic antioxidants can be toxic. Natural antioxidants, flavonoids are found to be the bioactive compounds in Flos sophorae, the flowers and buds of *Sophora japonica* L. [3]. Flos sophorae has been used to treat hematuria, hematemesis, hemorrhinia arteriosclerosis, and cerebral infarction [4]. Higher amounts of flavonoids were isolated from Flos sophorae, such as rutin, quercetin, kaempferol, and isorhamnetin, which exhibit a wide range of anti-inflammatory, antioxidant, radical scavenging, antibacterial, antiviral, antihyperglycemic, antiobesity, and antitumor [5,6] activities. Consequently, these compounds are usually selected as quality control markers for the quality evaluation of Flos sophorae and its preparations [7,8].

The type of method used for the extraction of bioactive compounds from medicinal plants can have a pronounced impact on the effects of the extract. Additionally, the solvent has a very high impact due to the solubility, ionization, or polarity of the analytes. IRAE is a novel, cost-effective, and environmentally friendly extraction technique that is capable of achieving high extraction efficiency over a short time with low levels of solvent and energy [9,10]. Compared with some traditional and modern extraction methods, IRAE provides considerable simple, fast, and economic advantages [11]. Furthermore, IRAE was recently used to prepare solid material, as well as being used to isolate anthocyanins, terpenoids, and phenolic acids from traditional Chinese medicines [12,13]. 

Previous studies have shown that the extraction efficiency of flavonoids is influenced by multiple parameters, including the extraction temperature, extraction time, solid to liquid ratio, and solvent concentration, as well as interactive effects associated with these different parameters [14,15]. Response surface methodology (RSM) is a powerful mathematical technique for optimizing the extraction of flavonoids [16]. To the best of our knowledge, there have been no reports in the literature pertaining to the use of response surface-optimized IRAE to optimize the extraction of the four flavonoids mentioned above from Flos sophorae. Although the extracts of Flos sophorae have strong antioxidant properties, the effect of the extraction method on the free radical scavenging ability of these extracts have never been investigated. 

Based on these considerations, the aim of this study was to optimize the conditions used for the IRAE of Flos sophorae to give an extract with a high yield of flavonoids that shows high antioxidant activity in vitro. A Box-Behnken design (BBD) was used in combination with a RSM to analyze the interaction effects of the different factors influencing the IRAE method. For comparison, we also investigated the extraction of Flos sophorae using ultrasound-assisted extraction (UAE) and heating reflux extraction (HRE). The results of this study therefore represent the optimum conditions for IRAE of Flos sophorae.

## 2. Results and Discussion

### 2.1. Single-Factor Experiment

The extraction conditions had a considerable impact on the extraction efficiency of the flavonoids. Previous experiments in this area focused on screening a wide range of different solvents and solid liquid ratios [17]. Furthermore, it is well known that the extraction time and operating costs are important factors that can have a significant effect on the efficiency of an extraction process. We, therefore, considered all of the factors that could affect the extraction yield of flavonoids obtained by IRAE.

#### 2.1.1. Extraction Solvents

**Figure 1:**
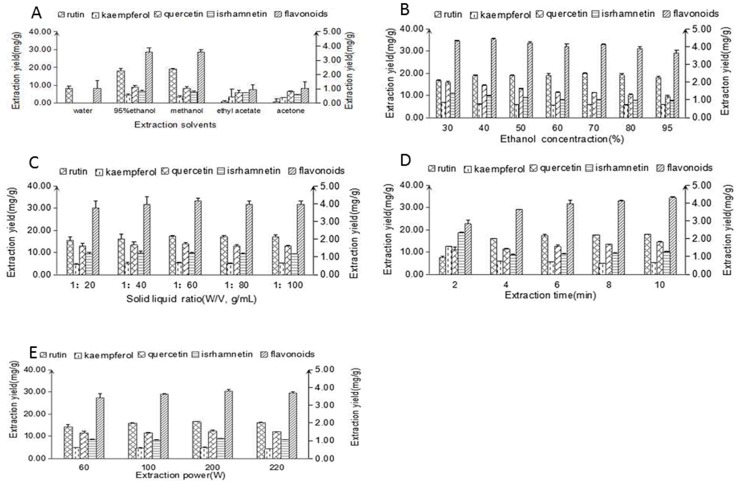
Effects of extraction solvents (**A**); ethanol concentrations (**B**); solid-liquid ratio (**C**); extraction time (**D**); and extraction powers (**E**) on the extraction yield of flavonoids (sum of the yield of rutin, quercetin, kaempferol, and isorhamnetin). Rutin, quercetin, and total flavonoids (left ordinate), kaempferol, isrhamnetin (right ordinate).

The nature of the extraction solvent has a very high impact due to the solubility, ionization, or polarity of the analytes. With this in mind, we screened several solvents in the current study, including deionized water, 95% (*v*/*v*) ethanol, methanol, ethyl acetate, and acetone, to determine the best solvent for the extraction of the flavonoids from Flos sophorae. As shown in Figure 1A, the extraction yields of methanol and 95% (*v*/*v*) ethanol were higher than those of all of the other solvents tested. Furthermore, the difference between the amounts of flavonoids obtained using either of these solvents was not significant (*p* > 0.05). Considering the toxicity of methanol, we proceeded to evaluate solutions composed of different ratios of ethanol and deionized water to reduce the amount of ethanol being used in the extraction solvent. 

Furthermore, we examined the use of aqueous solution containing 30–95% (*v*/*v*) ethanol to extract the compounds from Flos sophorae. As shown in Figure 1B, the extraction yield of the flavonoids gradually increased with increasing ethanol concentration and reached a maximum value at 40% (*v*/*v*) ethanol. Further increasing the ethanol concentration led to a slight decrease in the extraction yield. Based on this result, we selected an ethanol concentration of 40% (*v*/*v*) for extracting the flavonoids from Flos sophorae. This concentration range was consistent with the range reported by Wang’s group for the extraction of rutin from Flos sophorae [17].

#### 2.1.2. Solid-Liquid Ratio

The solid-liquid ratio is another crucial parameter which greatly influenced the extraction efficiency [18]. In this study, samples were extracted with solid–liquid ratio of 1:20, 1:40, 1:60, 1:80, and 1:100 (*w*/*v*, g/mL), respectively, when the other three conditions (ethanol concentration, extraction time, and extraction power) were set at 40%, 8 min, and 200 W, respectively. As shown in Figure 1C, increasing the solid to liquid ratio from 1:20 to 1:60 (*w*/*v*, g/mL) led to an increase in the extraction yield of the flavonoids. Since a high solid–liquid ratio prolonged the distance of diffusion toward the interior tissues. Further increasing the solid to liquid ratio beyond 1:60 led to a decrease in the yield of the flavonoids when ethanol was used as the solvent. Thus, a solid to liquid ratio in the range of 1:40–1:80 (*w*/*v*, g/mL) was selected as optimal for the following experiments. It is noteworthy that Xu et al. adopted the same solid to liquid ratio during their investigation of the extraction of rutin from Flos sophorae using cellulose [19].

#### 2.1.3. Extraction Time

Extraction time is an important parameter in any extraction process. Long extraction times can lead to unnecessary wasting or the decomposition of specific components, whereas short extraction times can lead to incomplete extraction processes. To investigate the effect of different extraction times on extraction yield of flavonoids, the extraction process was carried out using different times (2, 4, 6, 8, and 10 min), while other extraction parameters were fixed as follows: ethanol concentration 40%, solid–liquid ratio of 1:60 (g/mL), and infrared power of 200 W. The results of these experiments are shown in Figure 1D. The extraction yield increased considerably when the extraction time was increased from 2 to 10 min, and the highest yield was obtained at 10 min. This result was consistent with the results of Huang et al. [20] and Li et al. [21]. Taken together, these results suggested that an extraction time of 6–10 min would be suitable for the extraction of the flavonoids. 

#### 2.1.4. Extraction Power 

The effect of the infrared power on the yield of the flavonoids was investigated for powers of 60, 100, 200, and 220 W shown in Figure 1E, when other extraction conditions were fitted as follows: ethanol concentration of 40%, solid–liquid ratio of 1:60 (g/mL), and extraction time of 10 min. These results also showed that the yield of the flavonoids increased with increasing power and reached its maximum value at 200 W, which indicated that the yield of flavonoids started to maintain a dynamic equilibrium with increasing infrared power. This phenomenon is probably because the plant cells have already been ruptured and flavonoids have already been sufficiently extracted [11,21]. Based on these experiments results, a power of 200 W was selected for the subsequent experiments.

### 2.2. Response Surface Methodology (RSM) Experiment

#### 2.2.1. Box–Behnken Design (BBD)

BBD-RSM optimization is more advantageous than the single factor optimization due to being less time-consuming and less material-consuming. Additionally, compared to other **s**tatistical methods, such as orthogonal design [22] and uniform design [23], multivariate data can be fitted to linear or quadratic with interaction terms, which can be more accurately analyzed to explore the relationship between various factors and the effect of dependent variable [24]. 

On the basis of the single-factor experiments, the coded level for the three factors used in the BBD were selected as follows: ethanol concentrations (30, 40, and 50%), extraction time (6, 8, and 10 min), and solid liquid ratio (1:40, 1:60, and 1:80). Previous reports on the extraction of flavonoids from Flos sophorae also evaluated the influence of the ethanol concentration, extraction time, and solid–liquid ratio on the extraction yield [25,26]. A total of 17 experimental runs were performed according to the BBD matrix in Table 1 and the averaged values were used in the subsequent data analysis steps. 

#### 2.2.2. Fitting Model

The statistical significance of the polynomial equation was checked using Fischer’s F-test. The ANOVA data for the response surface quadratic model are shown in Table 2. The significance properties of the model terms were checked based on their respective *p*-values. The model was found to be highly significant, as evidenced by the results of an F-test, which gave an F-value of 6.31 (*p* < 0.05). The determination coefficient (R^2^) value (0.8903) suggested good correlation between the experimental and predicted values, and 89.03% variability of the response could be explained by the model. The F-value (5.38) and *p*-value (0.0688) for “lack-of-fit” indicated that the “lack-of-fit” was insignificant relative to the pure error. These data also indicated that the model equation was adequate for predicting response under any combination of the variables. It was, therefore, concluded from Table 2 that the coefficients of the linear and quadratic effects of each model term (*X*_2_, *X*_3_, *X*_1_^2^, and *X*_1_*X*_2_) were significant at the level of *p* < 0.05. The regression coefficients of the intercept, linear, quadratic and interaction terms of the model were calculated using the least square technique, and the results are shown in Table 2. Multiple regression analysis of the experimental data yielded the following second-order polynomial Equation (1):

*Y* = 32.21 + 1.26 *X*_1_ + 1.38 *X*_2_ + 1.33 *X*_3_ − *2.95 X*_1_*X*_2_ − 0.71 *X*_1_*X*_3_ + 0.67 *X*_2_*X*_3_ − 2.86 *X*_1_^2^ − 1.64 *X*_2_^2^ + 1.08 *X*_3_^2^ (1)

#### 2.2.3. Analysis of Response Surfaces

**Figure 2:**
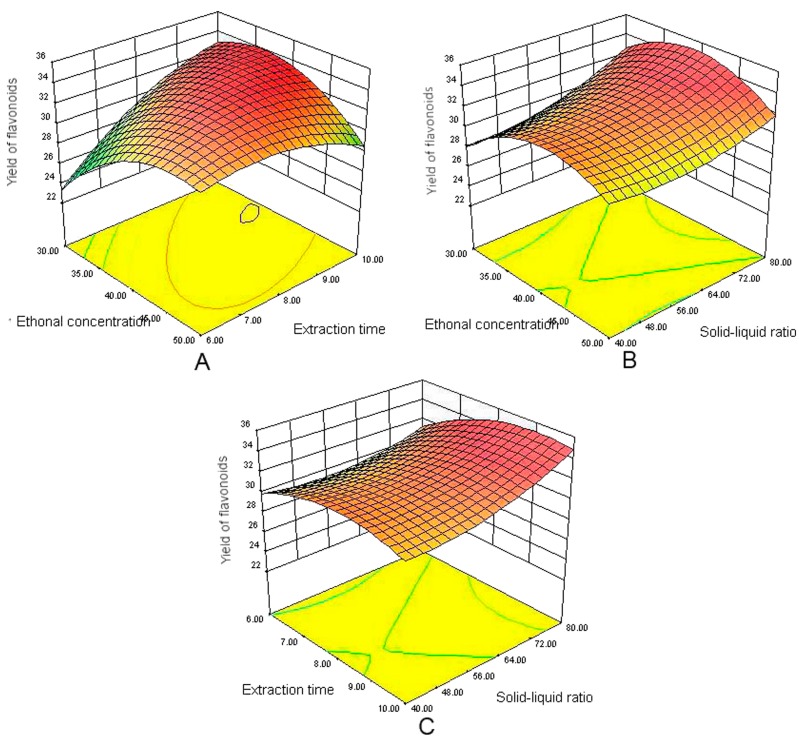
(**A**) Response surface graph showing interaction between ethanol concentration (%) and extraction time (min); (**B**) response surface graph showing interaction between ethanol concentration (%) and solid-liquid ratio; and (**C**) response surface graph showing the interaction between extraction time (min) and solid-liquid ratio (g/mL).

Three-dimensional plots give a comprehensive picture of the behavior of the prediction variances throughout a region and, hence, of the quality of the predicted responses obtained with BBD design [27]. Figure 2A shows the interaction effects between the ethanol concentration and the extraction time. These results indicated that the use of a high ethanol concentration in combination with an intermediate extraction time would lead to an increase in the extraction yield. No significant interaction effects were observed between the ethanol concentration and solid-liquid ratio, as shown in Figure 2B. The extraction yield initially increased with increasing ethanol concentration in high ethanol concentrations, but further increases in the ethanol concentration resulted in a reversal of this trend. As shown in Figure 2C, an increase in the extraction yield of flavonoids were achieved with the increases of extraction time and solid-liquid ratio. However, the yield no longer increases when the independent variables exceeded certain values.

#### 2.2.4. Verification of the Models

The optimum conditions for the selected parameters were predicted using the design expert software. The maximum predicted extraction yield was achieved for ethanol concentration, extraction time, and solid to liquid ratio values of 40% (*v*/*v*), 9 min, and 1:72 (*w*/*v*), respectively. Under these conditions, the predicted response for the purification factor was determined to be 33.817 mg/g. The predicted conditions were validated by conducting an experiment under the predicted conditions. The results revealed that an extraction yield of 33.199 ± 0.24 mg/g (average of three replicates) was obtained under the optimized conditions, which is very close to the model-predicted value.

### 2.3. Method Development and Validation

The developed ultra-high performance liquid chromatography (UHPLC) method can separate rutin (1.348 min), quercetin (3.204 min), kaempferol (3.666 min), and isorhamnetin (3.799 min) in a short time (5 min) by a gradient of 0.1% (*v*/*v*) formic acid aqueous solution and acetonitrile in the mobile phase.

The newly-developed UHPLC approach was evaluated in terms of its linearity, reproducibility, limit of detection (LOD), limit of quantification (LOQ), and extraction efficiency characteristics for rutin, quercetin, kaempferol, and isorhamnetin using spiked samples. Calibration curves of these four standard compounds were constructed from peak areas versus compound amounts. The LOQ and LOD for each compound were determined at signal-to-noise ratios (S/N) of 10:1 and 3:1. As summarized in Table 3, the linear ranges for rutin, quercetin, kaempferol, and isorhamnetin were 40.8–612.0, 4.6–149.5, 1.09–19.62, and 1.02–122.4 μg/mL, respectively. The correlation coefficients for the regression lines were in the range of 0.9994–0.9998, whereas the LOD values were in the range of 0.049–0.1061 μg/mL. 

The results for the precision, repeatability, and recovery rate characteristics are shown in Table 4. The precision of our newly-developed method was determined based on its intra-day (the daily) and inter-day (day-to-day) variability properties. The repeatability was found to be acceptable with a relative standard deviation (RSD) of less than 2% (RSD values: 0.74, 1.83, 1.56, and 1.43 for rutin, quercetin, kaempferol, and isorhamnetin, respectively). These values were obtained on the same day to assess the intra-day precision (*n* = 6) or over a three day period to assess the inter-day precision. The recovery rate was tested using the standard addition method, where the amount of four flavonoid standards (2.100, 2.642, 0.091, 0.160 mg), which was 50% of the amount in the extraction sample, was added to the sample (Table 4), and the contents of the resulting mixtures were analyzed. The mean values for the recovery rates of the four flavonoids ranged from 97.78 to 102.94% with RSD values of less than 4.2%. Taken together, these results demonstrate that our newly-developed method is both sensitive and precise, highlighting it suitability for the analysis of the Flos sophorae.

### 2.4. Comparison with Other Extraction Methods

The extraction yields of the flavonoids for the different extraction methods were compared, together with the antioxidant activities of the corresponding extracts, and the results are shown in Table 5 and Table 6. The optimum IRAE process showed several advantages over the other extraction methods evaluated in the current study. As shown in Table 5, the yield of the flavonoids obtained under the optimum IRAE conditions was 33.199 ± 0.24 mg/g, which was slightly higher than that of ultrasonic-assisted extraction (UAE). Notably, the extraction time of the IRAE method was just 10 min, making it much shorter than that of the UAE method, which required 30 min [28]. In addition, the yield of the flavonoids obtained using the IRAE method was obviously higher than that of the traditional heating reflux extraction (HRE) [29], which gave a yield of 20.112 ± 0.76 mg/g following an extraction time of 120 min at 80 °C.

As shown in Table 6, the Flos sophorae extract exhibited concentration-dependent responses in the DPPH and AAPH scavenging activity assays. The DPPH and AAPH scavenging capacities correlated well with the extraction yields of the flavonoids. In the DPPH assay, the IC_50_ value of the IRAE extract was 53.44 ± 0.01 μg/mL, followed by the UAE and HRE methods, which gave IC_50_ values of 72.03 ± 0.01 and 88.85 ± 0.01 μg/mL, respectively. The ORAC values for the IRAE, UAE, and HRE extracts were 3785.827 ± 52, 2983.864 ± 23, and 2420.291 ± 35 μmol/g, respectively. These results clearly show that all of the extracts exhibited significant antioxidant activity. Notably, the IRAE extract produced much better activity then the UAE and HRE extracts.

## 3. Experimental and Section 

### 3.1. Chemicals and Materials

Standards for rutin, quercetin, kaempferol, and isorhamnetin were purchased from Chengdu Must Bio-technology Co., Ltd. (Chengdu, China). Chromatographic grade methanol and acetonitrile were purchased from Fisher Corporation (Pittsburgh, PA, USA). Fluorescein disodium salt, trolox, 2,2-diphenyl-1-picrylhydrazyl (DPPH), and 2,2′-azobis (2-methyl-propionamidine) dihydrochloride (AAPH) were purchased from Sigma (St. Louis, MO, USA). All of the other reagents were purchased as the analytical grade from Beijing Chemical Reagent (Beijing, China). Purified water was obtained from a Milli-Q water purification system (Millipore, Molsheim, France). Flos sophorae (Hebei, China) were purchased from Beijing Tong Ren Tang Co., Ltd. (Beijing, China) and identified by Professor Bin Yang (Institute of Chinese Materia Medica, China Academy of Chinese Medical Sciences). All of the samples were dried, crushed, mixed, and passed through a 40-mesh sieve.

### 3.2. Ultrasound-Assisted Extraction 

Powered Flos sophorae (0.25 g) was weighed into a 100-mL flask and extracted with 50 mL of 40% (*v*/*v*) ethanol for 30 min under ultrasonic irradiation (200 W, 100 Hz) at room temperature, After the extraction, the mixture was filtered and analyzed by UHPLC to determine its flavonoid content, as described in the previous chapter.

### 3.3. Heating Reflux Extraction 

Powdered Flos sophorae (0.25 g) was placed in a 100-mL conical flask, followed by 50 mL of 40% (*v*/*v*) ethanol, and the resulting mixture was extracted for 120 min in a water bath at 80 °C. The mixture was analyzed to determine its flavonoid content by the proposed UHPLC method. 

### 3.4. Infrared-Assisted Extraction

An infrared lamp ( Qiyi Lighting Co., Ltd., Zhejiang, China) was placed below a 100-mL flask containing 0.25 g of and a suitable extraction solvent, and the distance between the flask and the lamp was adjusted to about 1 cm. The mixture obtained from the IRAE was filtered through a 0.2 μm Milipore filter (Millipore Filter Corp., Bedford, MA, USA) before being analyzed quantitatively. The IRAE extraction conditions were optimized through a series of single-factor tests, followed by BBD experiments.

### 3.5. Experimental Design for Infrared-Assisted Extraction

To evaluate the effect of infrared treatment on extraction yields of the four major flavonoids from Flos sophorae, extraction solvent (deionized water, 95% (*v*/*v*) ethanol, methanol, ethyl acetate, and acetone), ethanol concentration (30%, 40%, 50%, 60%, 70%, 80%, 90%, 95%), solid liquid ratio (1:20, 1:40, 1:60, 1:80, 1:100 g/mL), extraction time (2, 4, 6, 8, 10 min) and infrared power (60, 100, 200, 220 W) were investigated as single factor variables in the experimental design.

A three-level (1, 0, +1) three-factor BBD combined with RSM was adopted to determine the best combination of extraction variables of infrared-assisted extractin. The variables were ethanol concentration (*v*/*v* %, *X*_1_), extraction time (min, *X*_2_) and solid liquid ratio (g/mL, *X*_3_), while the extraction yield of flavonoids (mg/g, *Y*) was taken as the response for the design experiments. In the BBD test, 17 experiments were employed to fit the full quadratic equation model. The selection and ranges of these extraction factors were based on the preliminary single-factor experimental data.

### 3.6. Identification and Quantification of Flavonoids

**Figure 3:**
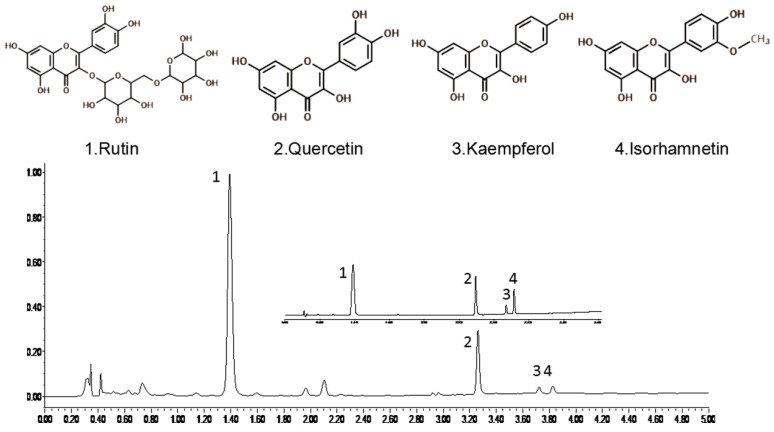
UHPLC chromatograms of rutin, quercetin, kaempferol, and isorhamnetin extracted from Flos sophorae samples by the IRAE method.

An Acquity ultra high-performance liquid chromatography system (Waters, Milford, MA, USA) consisting of a binary solvent manager, sample manager, and photodiode array detector (PDA) was used to analyze the flavonoids in the samples. The flavonoids were separated on an Acquity BEH C_18_ column (50 × 2.1 mm i.d., 1.7 μm, Waters). To obtain accurate and optimal chromatographic conditions, different UHPLC parameters were investigated, including mobile phases (methanol–water and acetonitrile—water with different modifiers, including formic acid and acetic acid), column temperatures (30, 35, 40, or 45 °C), and flow rates of the mobile phase (0.2, 0.3, 0.4, or 0.5 mL/min). The UV spectra of the compounds were detected at 256, 280, 356, and 360 nm by PDA detection. Finally, the optimized UHPLC condition was established by comparing the better baseline, shorter elution time, better resolution, and acceptable peak parameters for the marker compounds. The flow rate and injection volume were set as 0.4 mL/min and 1 μL, respectively. All of the chromatographic operations were carried out at 40 °C. The column was eluted using an aqueous solution of 0.1% (*v*/*v*) formic acid (solvent A) and acetonitrile (solvent B) under the following gradient elution conditions: 0–1.5 min (A: 85–80%), 1.5–2.5 min (A: 80–65%), 2.5–3 min (A: 65%), 3–4.5 min (A: 65–50%), and 4.5–5 min (A: 50–85%). The photodiode array detector was operated at 256 nm (Figure 3). The linearity, LOD, LOQ, precision, repeatability, and recovery rate characteristics of this method were evaluated to determine its reliability. The concentration of each flavonoid was determined based on an external standard method and expressed in units of milligrams per gram of dried sample. 

### 3.7. DPPH Radical Scavenging Assay

A DPPH assay was used to assess the radical scavenging activity of Flos sophorae using a slightly modified version of the method reported by Liu’s group [30]. Briefly, a 100 μL aliquot of Flos sophorae (diluted seven times in methanol) was mixed with 150 μL of a DPPH methanol solution (0.05 mg/mL). The resulting mixture was left to react in the dark for 30 min at 37 °C, and its absorbance was then read at a wavelength of 516 nm using a Thermo Scientific Microplate Reader. Methanol was used as a negative control. The DPPH free radical scavenging activity of each sample was calculated using Equation (2):

I (%) = [1 − (Ai − Aj)/Ac] × 100%(2)

where Ac is the absorbance of the DPPH solution without a sample; Ai is the absorbance of the test sample mixed with the DPPH solution; and Aj is the absorbance of the sample without the DPPH solution.

### 3.8. AAPH Radical Scavenging Assay

An ORAC assay was performed to evaluate the AAPH radical scavenging activity according to a modified version of the methods reported by the research groups of Jae-Hee and Hua Li [31,32]. Briefly, each well was charged with 100 μL dilutions of fluorescein sodium (8 × 10^−5^ mM). The wells were then charged with 50 μL sample solutions at different concentrations or 50 μL of phosphate buffer (75 mM, pH = 7.4) as a blank. The excitation and emission wavelengths were set at 492 and 514 nm, respectively. Each mixture was incubated at 37 °C for 10 min before being treated with 50 μL of AAPH (153 mM). The absorbance was recorded at 2 min intervals until the fluorescence decayed completely. The ORAC values were calculated based on a trolox (10–100 μM) linear regression equation and expressed as trolox equivalents (μmol trolox/g extract).

### 3.9. Statistical Analysis

All the experiments were performed in duplicate, and all results were reported by the average value ± SD (standard deviation). The experimental data were analyzed using Design Expert software (rial Version 8.0.6, Stat-Ease Inc., Minneapolis, MN, USA). ANOVA was performed to evaluate significant differences between the independent variables. The IC_50_ values were calculated using the SPSS software (Statistical Program for Social Sciences, SPSS Corporation, Chicago, IL, USA).

## 4. Conclusions

A novel and efficient IRAE method was employed to extract flavonoids from Flos sophorae. IRAE was performed with a three-variable, three-level Box–Behnken design based on the RSM. The experimental extraction yield of 33.199 ± 0.24 mg/g was obtained under the modified optimal IRAE conditions: ethanol concentration of 40% (*v*/*v*), extraction time of 9 min, solid to liquid ratio of 1:72, and an extraction power of 200 W. Compared with conventional HRE and UAE techniques, IRAE was a suitable technique to extract flavonoids from Flos sophorae, which exhibited a higher extraction yield with a shorter time. Furthermore, UHPLC analysis was developed for the identification and quantification of flavonoids in Flos sophorae. Moreover, the antioxidant activity assays demonstrated that the extract obtained by IRAE displayed a notable DPPH and AAPH radical scavenging ability in vitro. In a word, the overall findings of this study could be used to explore the potential applications of the flavonoids in Flos sophorae as a valuable source of natural antioxidants for industrial scale-up.

## Figures and Tables

**Table 1:** Response surface of the BBD and results for extraction yield of flavonoids from Flos sophorae.

Run	Ethanol Concentration (*X*_1_, %)	Extraction Time (*X*_2_, min)	Solid-Liquid Ratio (*X*_3_, g/mL)	Yield of Rutin (mg/g)	Yield of Quercetin (mg/g)	Yield of Kaempferol (mg/g)	Yield of Isorhamnetin (mg/g)	Yield of Flavonoids (mg/g)
1	30	10	60	17.11	12.16	0.49	1.17	30.94
2	50	8	40	17.57	10.62	0.50	1.04	29.73
3	40	8	60	17.02	12.53	0.58	1.20	31.33
4	40	8	60	17.95	13.36	0.60	1.22	33.13
5	50	10	60	16.86	10.62	0.46	1.01	28.95
6	40	10	40	16.46	12.68	0.53	1.16	30.83
7	40	10	80	18.10	13.59	0.60	1.24	33.53
8	40	6	40	17.62	11.81	0.56	1.11	31.10
9	50	8	80	19.21	11.43	0.54	1.07	32.25
10	30	8	40	14.56	11.07	0.44	1.09	27.17
11	30	8	80	16.46	14.13	0.64	1.31	32.55
12	50	6	60	18.9	9.97	0.55	0.97	30.39
13	40	6	80	17.96	11.47	0.62	1.10	31.14
14	40	8	60	17.35	12.47	0.55	1.16	31.53
15	40	8	60	17.70	12.38	0.58	1.17	31.84
16	40	8	60	17.80	13.58	0.61	1.23	33.21
17	30	6	60	11.79	7.67	0.28	0.83	20.57

Yield of flavonoids: sum of yield of rutin, quercetin, kaempferol, and isorhamnetin.

**Table 2:** Analysis of variance (ANOVA) for response surface quadratic mode.

Source	Sum of Squares	df	Mean Square	F-Value	*p*-Value prob > F	Significant
Model	131.63	9	14.63	6.31	0.012	Significant
*X*_1_ (ethanol concentration)	12.73	1	12.73	5.49	0.0516	
*X*_2_ (extraction time)	15.26	1	15.26	6.59	0.0372	*
*X*_3_ (solid liquid ratio)	14.15	1	14.15	6.11	0.0428	*
*X*_1_ *X*_2_	34.87	1	34.87	15.05	0.0061	**
*X*_1_ *X*_3_	2.04	1	2.04	0.88	0.3788	
*X*_2_ *X*_3_	1.77	1	1.77	0.76	0.4113	
*X*_1_^2^	34.45	1	34.45	14.86	0.0062	**
*X*_2_^2^	11.26	1	11.26	4.86	0.0633	
*X*_3_^2^	4.89	1	4.89	2.11	0.1898	
Residual	16.22	7	2.32			
Lack of fit	13	3	4.33	5.38	0.0688	not Significant
Pure error	3.22	4	0.81			
Corrected total	147.86	16				

* Significant (*p* < 0.05); ** extremely significant (*p* < 0.01).

**Table 3:** Linear regression data, LOD, and LOQ of four flavonoids in Flos sophorae.

Analyte	Regression Equation	*r*	Rang (μg/mL)	LOD (μg/mL)	LOQ (μg/mL)
Quercetin	*y* = 10,237*x* − 8295.4	0.9994	4.60–149.50	0.097	0.339
Kaempferol	*y* = 9798.3*x* − 4322.7	0.9995	1.09–19.62	0.081	0.202
Rutin	y = 5298.0*x* + 4079.5	0.9995	40.80–612.00	0.106	0.508
Isorhamnetin	y = 8484.4*x* − 15,031	0.9998	1.02–122.40	0.049	0.292

*y*, peak area; *x*, concentration of the analytes (μg/mL); *r*, correlation coefficient of the equation.

**Table 4:** Precision, repeatability, and recovery rate of four flavonoids in Flos sophorae.

Analytes	Intraday Precision (RSD, %, *n* = 6)	Interday Precision (RSD, %, *n* = 3)	Repeatibility (RSD, %, *n* = 6)	Recovery Rate ± (RSD, %, *n* = 5)
Rutin	2.45	0.96	0.74	102.94 ± 1.80
Quercetin	3.44	1.41	1.83	102.73 ± 0.87
Kaempferol	1.24	1.82	1.56	100.68 ± 2.39
Isorhamnetin	1.82	1.26	1.43	97.78 ± 4.19

**Table 5:** Comparison of extraction yield of four flavonoids among different methods (mg/g, *n* = 3).

Extraction Method	Extraction Time (min)	Solvent Volume (mL)	Yied of Rutin	Yied of Quercetin	Yied of Kaempferol	Yied of Isorhamnetin	Yied of Flavonoids
HRE	120	50	18.980 ± 0.510	1.218 ± 0.166	0.050 ± 0.018	0.662 ± 0.066	20.112 ± 0.76 ^a^
UAE	30	50	14.051 ± 0.425	13.803 ± 0.233	0.829 ± 0.016	1.534 ± 0.017	30.217 ± 0.88 ^b^
IRAE	9	18	18.980 ± 0.140	12.518 ± 0.172	0.545 ± 0.009	1.156 ± 0.014	33.199 ± 0.24 ^c^

HRE: heating reflux extraction; UAE: ultrasonic-assisted extraction; IRAE: infrared-assisted extraction. Values within the same column with different letters are significantly different at *p* < 0.05. Data are expressed as means ± SD with triplicates.

**Table 6:** Comparison of extraction yield and antioxidant activity among different methods.

Extraction Method	Extraction Time (min)	Solvent Volume (mL)	Extraction Yield (mg/g)	IC_50_ for DPPH (μg/mL)	ORAC (μmol/g)
HRE	120	50	20.112 ± 0.76 ^a^	88.85 ± 0.01 ^a^	2420.291 ± 35 ^a^
UAE	30	50	30.217 ± 0.88 ^b^	72.03 ± 0.01 ^b^	2983.864 ± 23 ^b^
IRAE	8.6	18	33.199 ± 0.24 ^c^	53.44 ± 0.01 ^c^	3785.827 ± 52 ^c^

HRE: heating reflux extraction; UAE: ultrasonic-assisted extraction; IRAE: infrared-assisted extraction; ORAC: oxygen radical absorbance capacity. Values within the same column with different letters are significantly different at *p* < 0.05. Data are expressed as means ± SD with triplicates.
